# Behavioral and Histopathological Study of Changes in Spinal Cord Injured Rats Supplemented with *Spirulina platensis*

**DOI:** 10.1155/2014/871657

**Published:** 2014-07-24

**Authors:** Izzuddin Aziz, Muhammad Danial Che Ramli, Nurul Suraya Mohd Zain, Junedah Sanusi

**Affiliations:** Department of Anatomy, Faculty of Medicine, University of Malaya, 50603 Kuala Lumpur, Malaysia

## Abstract

Spinal cord injury (SCI) is a devastating disease that leads to permanent disability and causes great suffering. The resulting neurological dysfunction and paralysis is proportional to the severity of the trauma itself. Spirulina is widely used as a nutritional supplement due to its high protein and antioxidant content. In the present study, the protective effect of the Spirulina treatment on locomotor function and morphological damage after SCI was investigated. Seventy Sprague-Dawley (SD) rats were divided into three groups: Sham (laminectomy alone), Control (laminectomy with SCI), and Experimental (laminectomy with SCI +180 mg/kg per day* Spirulina platensis*). A laminectomy was performed at T12 and an Inox No.2 modified forceps was used to perform a partial crush injury on the spinal cord. The rats were then perfused at 3, 7, 14, 21, and 28 days after injury for morphological investigations. The injured rat spinal cord indicated a presence of hemorrhage, cavity, and necrosis. Pretreatment with Spirulina significantly improved the locomotor function and showed a significant reduction on the histological changes. The experimental results observed in this study suggest that treatment with* Spirulina platensis* possesses potential benefits in improving hind limb locomotor function and reducing morphological damage to the spinal cord.

## 1. Introduction

Spinal cord injury (SCI) is one of the leading clinical causes of disability in young adults for which no suitable natural remedies have been found for SCI management. As such, the exploration of novel therapeutic agents to enhance neuroprotection after SCI is needed [1]. The spinal cord does not have to be severed in order for loss of functioning to occur. In fact, in most people with SCI, the spinal cord is intact, but the damage to it results in loss of sensory or autonomic function and loss of normal motor function which may lead to paraplegia and quadriplegia. There are about 10,000 new SCI cases every year reported around the world, especially in the European countries. Males between the ages of 16 to 30 are among the majority who suffer from SCI and this occurs throughout the world with an annual incidence of 15 to 40 cases per million population [2].

There are some therapies available for SCI management such as erythropoietin, minocycline, inosine, riluzole, pioglitazone, and others [3]. At present, a high dose of methylprednisolone (MP) is the most common drug used for the treatment of spinal cord injury patients since the results of the landmark National Acute Spinal Cord Injury Studies (NASCIS) II trial in the 90s were published. However, many clinicians and scientists around the world have questioned the effectiveness of using MP due to conflicting results of experimental studies [4, 5] compared to the minor neurological improvements seen in patients [6, 7]. Furthermore, MP has been associated with certain side effects such as anxiety, dizziness, and mental depression and it can increase risk of infection both of wounds and at the site of trauma [8].

Spirulina is widely used as a nutritional supplement as it is complete with about sixty percent highly digestible protein, contains all the essential amino acids, and is rich in gamma-linolenic acid (GLA), minerals, trace elements, chlorophyll, and digestive and restriction enzymes [9]. In addition, it contains a wide range of antioxidants such as superoxide dismutase (SOD), provitamin-A (beta-carotene), vitamin C, E, selenium and phycocyanin, and flavonoids which have been proven in previous studies [10–15].

Spirulina is now attracting even more attention from medical scientists as it can be used as a nutraceutical and is a potential source of pharmaceuticals due to its ability to inhibit viral replication, strengthen both the cellular and humoral arms of the immune system, and aid in the regression and inhibition of cancer [16]. In addition to this, certain studies have reported that a dietary supplementation of Spirulina may reduce ischemic brain damage and provide a neuroprotective effect in cerebral ischemia-reperfusion injuries [17, 18]. This study is an initial attempt to investigate the effects of Spirulina supplementation on behavioral and morphological changes in traumatic injured rat spinal cords.

## 2. Materials and Methods

### 2.1. Animals

Seventy male Sprague-Dawley (SD) rats, weighing 200 ± 50 g, eight weeks old, were purchased from the Experimental Animal House, University of Malaya. All the rats were then divided into three groups: sham (laminectomy without SCI, *n* = 10), control (laminectomy with SCI, *n* = 30), and experimental (laminectomy with SCI +180 mg/kg per day* Spirulina platensis*, *n* = 30). For the control and experimental groups, all the rats were divided into five subgroups consisting of those sacrificed at 3, 7, 14, 21, and 28 postoperative days, respectively, with *n* = 6 for each group. The rats were kept at the standard conditions of temperature (23 ± 2°C) and humidity (50 ± 10%) with an alternating 12-hour light/dark cycle. The animals were kept in the Experimental Animal House, University of Malaya. The animals were acclimatized to the laboratory conditions prior to experimentation and the study was conducted according to the study protocol approved by the Animal Care and Use Committee (ACUC), Faculty of Medicine, University of Malaya, Kuala Lumpur, with the reference number ANA/27/01/2012/IA (R).

### 2.2. Surgical Procedures

All the rats were anesthetized using a mixture of 100 mg/kg of ketamil (100 mg/mL ketamine, Australia) and 10 mg/kg of xylazil (20 mg/mL xylazine, Australia) via intramuscular injection (IM). The fur overlying the thoracic vertebral column of the rat was removed using a shaver (Wahl, USA). The rats were then placed in a prone position on the operating table under sterile conditions. A midline dorsal incision was done on the rats and the paravertebral muscles were separated from the vertebrae. The vertebral was then removed with a microrongeurs to expose the underlying dura mater. A partial crush injury was extradurally performed for the control and experimental groups by compressing for 30 sec [19] at the twelfth thoracic spinal cord segment (T12) using Inox number 2 modified forceps (Dumont, Switzerland). The wounds were cleansed with saline and the muscle and connective tissue were closed with a 4-0 nylon. Finally, the skin was closed with surgical staples. The rats were then kept in the experimental animal house during recovery.

### 2.3. Postinjury Care

Each rat received 1 mL lactated ringers subcutaneously and 0.4 mg/kg Baytril (Bayer, Korea) intraperitoneally (IP) immediately after the surgery until day 7 following operation. The rats were carefully monitored for evidence of urinary tract infection or any sign of systemic disease. Additional Baytril was given for any evidence of hematuria/urinary infection. Immediately after the surgery, the animals exhibited hindlimb paralysis and a loss of bladder function. Following this, manual bladder expression was performed twice daily for the first week and once daily after that until the spontaneous emptying of the bladder had recovered. Animals were housed in individual cages with bedding.

### 2.4. Diet and Supplementation

All rats were fed with the standard rat chow. Animals were given access to food and water* ad libitum*. For the experimental group, the rats were supplemented with 180 mg/kg per day of* S. platensis* (Mutiara Saintifik, Kuala Lumpur) through an esophageal feeding tube starting from day 1 after the injury up to 3, 7, 14, 21, and 28 postoperative days according to their subgroup.

### 2.5. Behavioral Analysis

The right and left hindlimb locomotor functions were evaluated and graded using Basso, Beattie & Bresnahan's (BBB) locomotor rating score [20]. The locomotor evaluation was done in an open field 1.2 m diameter, circular, smooth-surfaced activity chamber for 4 mins. In this test, specific components of functional behavior were analyzed such as limb movement, paw placement/position, stepping, coordination, toe clearance, and tail position. A score of 0 was given when no spontaneous hindlimb movement was observed, while a score of 21 indicated normal locomotion. Hindlimb movement was scored by 2 investigators that were blinded to the experiment.

### 2.6. Histological Procedure

At the time of sacrifice, animals were overdose anesthetized with a mixture of 0.35 mL of ketamil (100 mg/mL ketamine, Australia) and 0.15 mL of xylazil (20 mg/mL xylazine, Australia). Then, the rat was perfused through the heart with 300 mL of normal saline followed by 300 mL of 4% paraformaldehyde. A 3 cm section including the T12 level of the spinal cord was then exposed and removed from the vertebral column and then fixed in the same fixative overnight. The tissue was embedded in paraffin and sectioned sagittally (4 *μ*m thickness). The sections were stained with Haematoxylin and Eosin (H and E) to help visualize the morphology of the spinal cord.

### 2.7. Quantitative Image Analysis Procedure

The H and E stained sections were used for image analysis using the NIS-Elements AR 3.2 software. The area containing the spinal cord lesions was captured through a camera (Nikon, Japan) connected to the microscope (Nikon eclipse 80*i*, Japan). The area of the lesion was distinguished from the surrounding normal spinal cord tissue by the presence of necrosis, inflammatory cells, cysts, or cavities and also the appearance of pale staining. The lesions were measured according to two parameters: the lesion size from rostral to caudal and the lesion size from lateral to lateral.

### 2.8. Statistical Analysis

All the data collected from the experiment was recorded and analyzed using SPSS software for Windows (version 16.0, Inc., Chicago, IL, USA). One-way analysis of variance (ANOVA) with Tukey's post hoc analysis was used to compare differences among the groups. In each test, the data was expressed as the mean value ± standard deviation (SD) and differences were considered to be significant at *P* < 0.05.

## 3. Results

### 3.1. Changes in Body Weight

**Figure 1:**
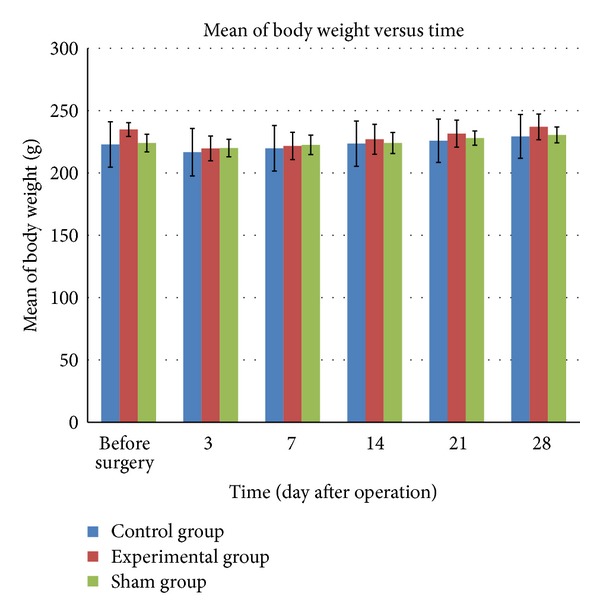
Changes in body weight in rats before and after surgery (postsurvival interval). The bar graphs indicate the mean, and the bars indicate SD of the measurements.

The body weight of all rats in the control, experimental, and also sham groups decreased immediately within 3 days after surgery (Figure 1). Although the rats showed a decrease in body weight after the surgery, this loss was not significantly different. On day 7 after operation onwards, all the rats demonstrated an increase in body weight and on day 28 after operation, the weight exceeded their presurgery weight.

### 3.2. Locomotor Function Assessment

**Figure 2:**
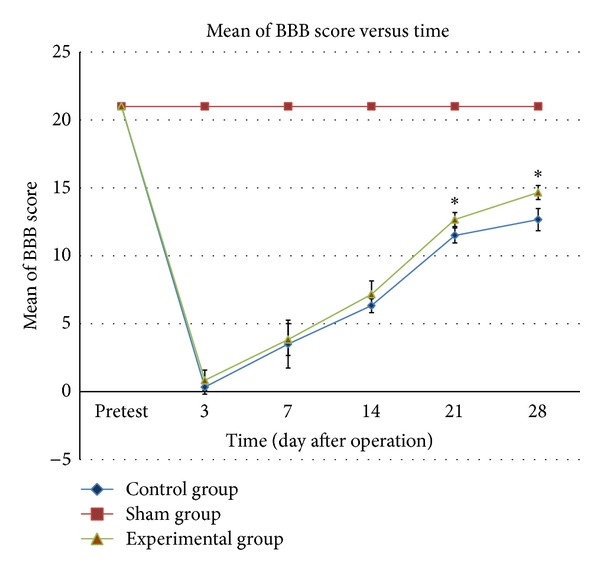
BBB score among the three groups of rats one day before surgery (pretest) and after surgery. The line graphs indicate mean of BBB score taken while the bars indicate SD of the measurements. ^∗^ *P* < 0.05 for experimental group versus control group.

All the rats in the control and experimental groups demonstrated signs of paraplegia but the rats then showed a slight improvement throughout the 4 weeks after the surgery. Spontaneous emptying of the bladder was recovered a week after the surgery. The locomotor function of all the rats that had undergone the laminectomy alone (i.e., the sham group) was found to be unaffected, as shown by the BBB score (a score of 21). Before the surgery, all the rats in the control group had normal BBB scores of 21. Three days immediately after surgery, the paralysis of their hindlimbs resulted in a reduction in the BBB score (Figure 2) but the rats then recovered widespread movement of their locomotor hindlimb joints, which meant that the BBB score increased up to day 28 after the surgery. The BBB score for all the rats in the experimental group also displayed a similar score pattern when the score was reduced at day 3 after operation and then increased at 7, 14, 21, and 28 days following operation. At days 21 and 28 after operation, all the rats demonstrated an improvement in their locomotor functions, during which the BBB score showed a significant difference between the control group and the experimental group. This study found that the BBB score for all the rats in the experimental group was higher compared to those in the control group at day 3 up to day 28 after operation.

### 3.3. Histopathology Study

**Figure 3:**
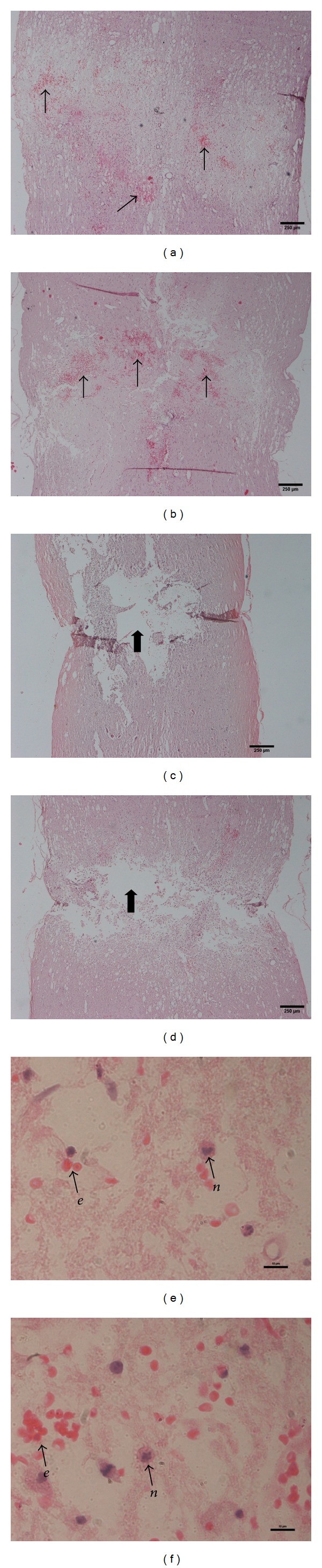
Histopathology slaids for control (a) and experimental (b) group 3 days after injury and control (c) and experimental (d) group 7 days after injury. The photographs illustrate H and E stained sections at the crush site. Arrows showed the area of hemorrhage that contains pockets of erythrocytes. Block arrows indicate cavity (empty lesion) with the cavity in the experimental group (d) being smaller than that in the control group (c). The difference of erythrocytes and neutrophils appearance in control (e) and experimental (f) group where (*e*) indicate erythrocyte while (*n*) indicate neutrophil. Scale bar, 250 *μ*m for panels (a)–(d) under ×4 magnification and 10 *μ*m for panels (e) and (f) under ×100 magnification.

A histopathology study done using H and E staining on the spinal cord tissue showed the appearance of lesions at the crush site in the control (Figure 3(a)) and experimental (Figure 3(b)) groups at day 3 after operation. Meanwhile, in the sham group, no lesions were observed in the tissue. The lesion area in the control and experimental groups was observed to lead to areas of progressive necrosis and cavitation (Figures 3(c) and 3(d)) which extended farther rostrally and caudally until day 7 following operation. At day 14 after operation onwards, the size of the cavity and lesion decreased for both control and experimental groups. The presence of hemorrhagic foci was observed in the middle of the crushed edge. In all the tissue observed, a number of erythrocytes and neutrophils (Figures 3(e) and 3(f)) had emerged at the primary site. There was a difference in the histological appearance in certain important aspects among the control and experimental groups. In this study, the increase in lesion size in the H and E stained sections for the experimental group was significantly less than that in the control group. The lesion size was small and was limited to the area adjacent to the crush site only while the presence of neutrophils and erythrocytes in this tissue was higher (Figure 3(f)). There were less necrosis and a smaller cavity (Figure 3(d)) along the dorsal column.

### 3.4. Quantitative Analysis

#### 3.4.1. Lesion Size from Rostral to Caudal

**Figure 4:**
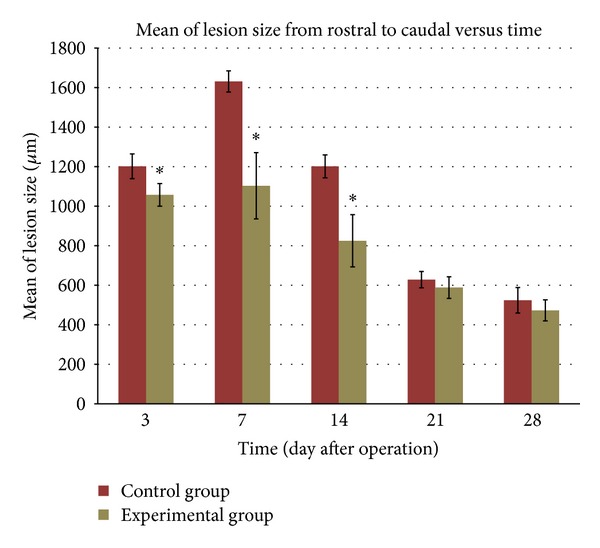
Effects of* S. platensis* on the lesion size from rostral to caudal after spinal cord injury (*n* = 6 per time point). The bar graphs represent mean of the measurement while the bars represent SD. ^∗^ *P* < 0.05 for experimental group versus control group.

A quantitative study revealed differences between the lesion size in the control and experimental groups throughout the postinjury survival interval (Figure 4). The lesion sizes in the animals supplemented with 180 mg/kg per day of* S. platensis* were significantly (*P* < 0.05) smaller compared to those in the control group when they were measured at day 3 after operation. The lesion size was found to have increased at day 7 following operation in both groups but the lesion sizes in the experimental group were significantly (*P* < 0.05) smaller compared to those in the control group. Image analysis showed that the lesion size was smaller starting from day 14 onwards in both the control and experimental groups. The measurement taken from the experimental groups showed the efficacy of* S. platensis* in reducing neurological deficit when there was a significantly (*P* < 0.05) smaller lesion size in the animals supplemented with* S. platensis* at days 3, 7, and 14 compared to those that were not supplemented with it.

#### 3.4.2. Lesion Size from Lateral to Lateral

**Figure 5:**
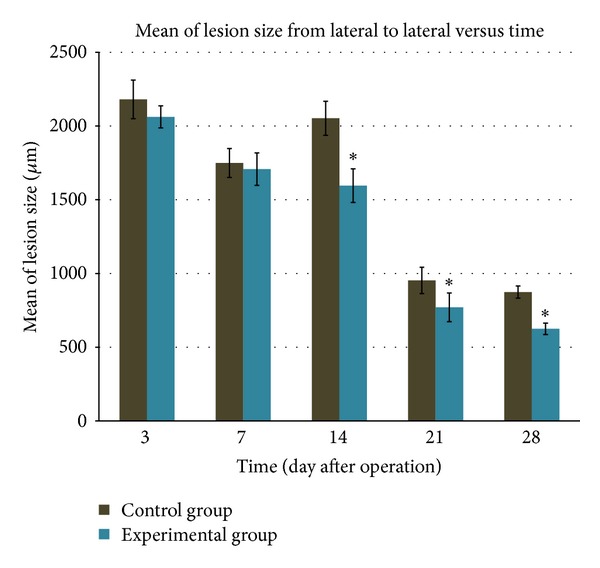
Difference in lesion size from lateral to lateral between animals without supplementation of* S. platensis* (control group) and animals supplemented with* S. platensis* (experimental group). The bar graphs indicate mean while the bars indicate SD of the measurement. ^∗^ *P* < 0.05 for experimental group versus control group.

From the analysis of the spinal cord tissue, a difference of the lesion size when measured from lateral to lateral end was found (Figure 5). The tissue was ruptured in both the experimental and control groups at day 3 following operation but the lesion size measured in the experimental group was smaller compared to the control group, even though it was not significant. At 7th postoperative day, there was a reduction in lesion size but the difference of the size in the control and experimental group was not significant. The lesion size in the experimental group was significantly smaller compared to the control group at days 14, 21, and 28 after surgery.

## 4. Discussion

*S. platensis* at a dose of 180 mg/kg per day as used in this study showed its capacity to reduce the neurological deficits in the spinal cord injured rats. This dose is equivalent to the normal human dose because the normal dose of* S. platensis* taken by human is essentially around 500 to 2000 mg per day. Moreover, a previous study [18] proved that* S. platensis* at a dose of 180 mg/kg per day showed a significant reduction in cerebral neurological deficits.

In the present study, we tested whether the supplementation of* S. platensis* in the traumatic injured rat spinal cord has a protective effect. The current study is the first to investigate the effects of* S. platensis* on traumatic SCI. Based on the findings that have been demonstrated by both the behavioral and histopathological outcomes, the most significant finding in this study is that* S. platensis* possesses potential benefits in improving locomotor function and reducing morphological damage to the spinal cord. Many techniques have been developed by researchers and scientists around the world to induce crushes on SCI models. The most commonly used model is the contusion model [21–23]. This induces a SCI by weight drop [24] and an impactor rod [25]. In addition to this, the compression model has also been used in SCI research by dropping weight [26], balloon angioplasty catheters [27], and also cerebral vascular clips [28].

In this study, we used a pair of forceps to induce a crush injury to the spinal cord as it is easy to use, is affordable, and proved to be clinically relevant. From our study, we found that even with using forceps, the spinal cord crush still produced proportional tissue loss, necrosis, cavitation, and corresponding locomotor function deficits. Our results are similar to the findings of researchers who have also used forceps to induce crushes in SCI models and reported to perfectly reflect the pathological and physiological features of SCI [29, 30].

Body weight changes in this study were analyzed as it is a general indication of health. From the observations of this study, all animals in the sham, control, and also experimental groups which had undergone the surgical procedure had a slight drop in their body weight 3 days after surgery. This weight loss might be induced by trauma and loss of fluid during the surgical procedure [6].

From our functional study, the locomotor function assessment revealed significant (*P* < 0.05) neurological deficits in SCI rats. The rats become paralyzed immediately after the SCI procedure and neurological recovery was observed after one week. The BBB locomotor rating scale [31] was employed in this study as it has been shown to produce reproducible results and remains the gold standard in evaluating the functional assessment of the spinal cord injury model. The results of the BBB score obtained from this study indicated that* S. platensis* supplementation prevented neurological deficits in the SCI rats. This was proved by the BBB score analysis which showed that the BBB score in the experimental group was significantly higher (*P* < 0.05) than that in the control group at days 21 and 28 after operation. As such, the behavioral assessment supported the beneficial effect of the* S. platensis* supplementation.

The morphological study was further analyzed as it supported the functional study outcomes. The spinal cord undergoes a sequence of pathological changes after a traumatic injury that causes the appearance of edema, hemorrhage, neuronal necrosis, axonal necrosis, demyelination, cyst formation, and also cavitation [32–34]. The histopathological outcomes in the animals in the control group in this study which had undergone the crush injury showed a presence of lesions in the tissue and there was an extended area of hemorrhage along the dorsal length of the cord. After a week, the crush site and the surrounding hemorrhagic zone had developed to progressive necrosis and cavitation. This finding was comparable with that of previous studies which have been well-characterized in rats [30, 35–37]. Furthermore, a previous study by Ducker et al. [38] also showed that the pathological changes worsened with time when necrosis is found in the tissue 6 days after an injury, which is quite similar to what we found at day 7 after the injury. The present study showed areas of empty lesions in the tissue where the large cavities developed are bordered by glial connective tissue scarring. This is similar to the results in the previous study conducted by Fujiki et al. and Zhang et al. indicating that a progressive series of cavitation resulted in an empty lesion [39, 40].

The presence of leukocytes in the spinal cord injury tissue represents the pathological changes towards the injury. From the microscopic observation in this study, we found that neutrophils are present at the injury site in response to the trauma. This observation is consistent with a previous study [41], where neutrophils accumulated at the site of compression. The accumulation of neutrophils in the areas of the hemorrhagic zone after a spinal cord injury is believed to act as phagocytes of red blood cells and necrotic tissue [41, 42]. Neutrophils play a vital role in secondary injury processes in traumatic injured rat spinal cords as the interaction between activated neutrophils and endothelial cells leads to spinal cord injury. These activated neutrophils release reactive oxygen species that can damage endothelial cells. This means that both the reactive oxygen species and the neutrophils are the main components in damaging the endothelial cells involved in the pathological changes which then result in the spinal cord injury. In this study, even though the* S. platensis* did not inhibit the accumulation of the neutrophils, it is not the only main feature as a new strategy for reducing the severity of spinal cord injury. To date, MP is still being used as a pharmacological treatment of spinal cord injury patients even though it does not inhibit neutrophil activation [43].

From the quantitative analysis, we found that* S. platensis* showed its efficacy to inhibit the morphological damage in the spinal cord tissue.* S. platensis* has been proven to be a neuroprotective agent in cerebral-ischemia injury rats [17, 18], but no previous research has tested it in rats with spinal cord injuries. In this study, we found that the lesion and cavity size remained smaller in the experimental group compared to the control group throughout the period following the injury. The* S. platensis* supplementation in this study demonstrated its capacity to reduce neurological deficits in the spinal cord. We found that the pattern of recovery in both control and experimental groups was the same but the size of the lesion and cavity in the experimental group was smaller than that in the control group, which indicate that* S. platensis* may inhibit the neurological deficits in the rat from that particular group. The reduction of lesion and cavity size is among indicators of the beneficial effects of the treatment, as was demonstrated in a previous study [30], which also reported that a reduction in lesion and cavity size was seen in the treated group.

## 5. Conclusion

The supplementation of* S. platensis* may inhibit progressive damage on the spinal cord injury tissue and can improve hindlimb locomotor function in traumatic injured rat spinal cords. More research is needed to confirm the efficacy of* S. platensis* in spinal cord injury studies as it may offer alternatives to SCI management.
